# Implementing a nurse-enabled, integrated, shared-care model involving specialists and general practitioners in early breast cancer post-treatment follow-up (EMINENT): a single-centre, open-label, phase 2, parallel-group, pilot, randomised, controlled trial

**DOI:** 10.1016/j.eclinm.2025.103090

**Published:** 2025-02-05

**Authors:** Raymond J. Chan, Fiona Crawford-Williams, Chad Yixian Han, Lee Jones, Alexandre Chan, Daniel McKavanagh, Marissa Ryan, Christine Carrington, Rebecca L. Packer, Megan Crichton, Nicolas H. Hart, Emma McKinnell, Melissa Gosper, Juanita Ryan, Bethany Crowe, Ria Joseph, Carolyn Ee, Jane Lee, Steven M. McPhail, Katharine Cuff, Laisa Teleni, Jon Emery

**Affiliations:** a Caring Futures Institute, College of Nursing and Health Sciences, Flinders University, Adelaide, South Australia, Australia; b Cancer and Palliative Care Outcomes Centre, School of Nursing, Queensland University of Technology, Brisbane, QLD, Australia; c Princess Alexandra Hospital, Metro South Health, Brisbane, QLD, Australia; d QIMR Berghofer Medical Research Institute, Herston, QLD, Australia; e Department of Clinical Pharmacy Practice, School of Pharmacy and Pharmaceutical Sciences, University of California, Irvine, USA; f School of Health and Rehabilitation Sciences, The University of Queensland, Brisbane, QLD, Australia; g Human Performance Research Centre, INSIGHT Research Institute, University of Technology Sydney (UTS), Sydney, NSW, Australia; h Exercise Medicine Research Institute, School of Medical and Health Sciences, Edith Cowan University, Perth, WA, Australia; i Institute for Health Research, The University of Notre Dame Australia, Perth, WA, Australia; j Department of General Practice and Primary Care, University of Melbourne, Melbourne, VIC, Australia; k Centre for Cancer Research, University of Melbourne, Melbourne, VIC, Australia

**Keywords:** Breast cancer, Models of care, Nurse-led, Shared-care, Survivorship, Quality of life

## Abstract

**Background:**

Current models of post-treatment cancer care rely heavily on hospital-based, medical specialists and do not sufficiently leverage primary care. Many breast cancer survivors face ongoing unmet needs that may benefit from a multidisciplinary, shared-care approach. We aimed to evaluate the feasibility and preliminary effectiveness of implementing nurse-enabled, shared-follow-up care between the acute and primary care setting for early-stage breast cancer.

**Methods:**

In this single-centre, open-label, phase II, pilot, randomised, controlled trial, individuals diagnosed with breast cancer (Stage 0–III) were randomised 1:1 to either usual care or intervention, which includes a 1) Specialist Nurse Consultation to co-develop a survivorship care plan (SCP), 2) Pharmacist Consultation, 3) Case Conference with General Practitioner (GP), and 4) shared follow-up care arrangements. Feasibility and effectiveness outcome measures, including health-related quality of life (primary outcome), physical activity and nutrition, patient experience, and financial toxicity were collected at baseline, and at 3-, 6-, and 12-months, with health service utilisation data at 24-months. Bivariate and multivariable, intention-to-treat analyses were conducted. This trial is registered at Anzctr.org.au (ACTRN12619001594112).

**Findings:**

From 3rd December 2019 to 13th April 2021, 61 participants were randomised (intervention n = 29; usual care n = 32); mean age 62.9 standard deviation (SD) = 10.9 years. The intervention was feasible with 100% completion rates across all elements of the specialist nurse consultation and GP case conference. Evaluation of the 28 SCPs indicated the top three goals were exercise (n = 23), diet (n = 12) and mental well-being (n = 11). All care goals can be supported by GPs. No differences were observed between groups for health-related quality of life and the other effectiveness outcomes measures listed above at all timepoints (P > 0.05 for all). There were significantly fewer average post-treatment radiation oncology appointments per patient in the intervention group compared to the control group (0.69 versus 1.27, P = 0.013) at 24-months. Number of unplanned hospital presentations at 24-months were low across both intervention (n = 7) and control (n = 4) groups.

**Interpretation:**

Nurse-enabled, shared-care arrangements for women with early-stage breast cancer is feasible, and is as safe as specialist-led model of care. It may provide a more sustainable model of care in a longer term. GPs can meet the survivorship care needs identified breast cancer survivors. This trial can inform a large, pragmatic, hybrid effectiveness-implementation trial.

**Funding:**

Metro South Health Research Support Scheme Project Grant.

**Research in context**

**Evidence before this study**

We searched PubMed for clinical trials evaluating nurse-enabled shared care in patients with early breast cancers published from inception to Jan 10, 2025, using search terms “Breast Neoplasms” or “Breast cancer” and “shared care.” Out of the 41 results, one randomised controlled trial (RCT) (ACTRN 12606000022561) looked at shared care during chemotherapy treatment, for patients with cancer that included breast cancer. No prospective or pilot RCTs were conducted to test whether nurse-enabled shared-care model involving specialists and general practitioners in early breast cancer post-treatment was feasible or could improve outcomes in patients with early-stage breast cancer at post-treatment follow-up phase.

**Added value of this study**

This is the first pilot randomised controlled trial demonstrating that a specialist cancer nurse-enabled shared-care model between treating medical specialists and general practitioners for women with early-stage breast cancer in Australia is feasible and warrants further investigation with a larger RCT.

**Implications of all the available evidence**

Findings from the present study indicate strong feasibility of implementing a nurse-enabled, shared-care, cancer survivorship care model for women with early-stage breast cancer, which is as safe and potentially as effective as a treating medical specialist-led model of care. Although this intent of this study was *not* to sufficiently powered to detect differences in health-related quality of life and other effectiveness outcomes, the model of care under testing was comprehensive and complex in nature, involving multidisciplinary team members across the acute and primary care settings. Our findings indicate that all care goals identified by survivors during this follow-up period study align with the role of the GP and community care providers. A shift of focus from acute cancer care to a shared-care arrangement in the post-treatment phase may enhance future sustainability of the system.

## Introduction

Breast cancer is the second most diagnosed cancer in the world, and the most common cancer in women.^1^ In Australia, an estimated 20,428 new cases were diagnosed in 2022.^2^ Improvements in screening and early detection, as well as accessibility of effective treatments, has increased the 5-year relative survival rate of breast cancer in developed countries.^1^ Although survival rates are high, breast cancer survivors deal with a myriad of acute- and long-term adverse consequences^3^ including acute and chronic pain and fatigue, lymphedema, insomnia, early menopause, sexual dysfunction, infertility, weight issues, financial toxicity, anxiety, depression and secondary malignancies following cancer treatment.^4^^,^^5^ It is critical to address the complex post-treatment needs for people diagnosed with breast cancer using a comprehensive, integrated, person-centred model of care.^6^

In Australia, the predominant model of post-treatment follow-up care for early breast cancer is treating medical specialist-led and primarily medically driven, which focuses on surveillance for disease recurrence and symptom management. However, such models of care are unsustainable with ever-increasing numbers of new breast cancer diagnoses, that may render such models of care insufficient to meet the care needs of breast cancer survivors and their families.^7^ To overcome these issues, international guidelines,^7^ Australian Optimal Cancer Care Pathways,^8^ and Australian government guidelines,^9^ recommend an integrated, shared-care model that involves both cancer specialists and primary care providers as partners in the follow-up care of early breast cancer.^10^

Shared-care models have been reported as feasible, acceptable, and safe, and may provide a more person-centred and cost-effective approach than current models of survivorship care led by treating medical specialists.^11^^,^^12^ Emerging shared-care models have been tested in Australia with some success.^13^^,^^14^ Two recent Australian trials reported that shared-care is as effective as specialist-led care, however potentially more cost-effective and satisfactory to the prostate and colorectal cancer survivors evaluated.^13^^,^^14^ Despite promising evidence and advocacy from national governing bodies and international experts, shared-care models have not been routinely implemented in practice and a range of a barriers to implementation exist.^15^ To overcome key barriers to implementation and ensure seamless coordination between primary and acute care settings within these shared-care models, a central coordinator such as a specialist cancer nurse (SCN) responsible for care planning, communication, advising cancer survivors of the benefits of shared-care and negotiation with GPs is paramount.^16^

The objective of this study was to test the feasibility, process outcomes, and preliminary effectiveness of a nurse-enabled, integrated, shared-care model of follow-up care for early breast cancer, involving cancer specialists and General Practitioners (GP) and the wider care team in post-treatment care, compared to usual specialist-led models.

## Methods

### Study design

A single-centre, open-label, phase 2, pilot, randomised controlled trial (RCT) was conducted.^17^

Detailed descriptions of the trial protocol including the nurse-enabled, integrated, shared-care model involving specialists and GPs in early breast cancer post-treatment (EMINENT) intervention have been published previously,^17^ and are included in supplementary file–protocol. This trial was conducted at a metropolitan tertiary teaching hospital (Princess Alexandra Hospital (PAH), Brisbane, Australia) between 3rd December 2019 to 13th April 2021, and reported in accordance with the Consolidated Standards of Reporting Trials (CONSORT) 2022 guidance statement for pilot or feasibility trials, the CONSORT 2017 guidance statement for reporting nonpharmacological trials, and the CONSORT 2022 guidance statement for reporting harms. This trial is registered at Anzctr.org.au (ACTRN12619001594112).

### Ethics

Ethics approval was obtained from the Metro South Hospital and Health Services Human Research Ethics Committee (HREC/2019/QMS/51956). All participants provided written informed consent.

### Participants

Participants were eligible if they were at least 18 years of age and had a diagnosis of curable early breast cancer (i.e., no distant metastases). They must have been receiving care at the Princess Alexandra Hospital within a specified time frame: from four weeks before completion to 18 months after completion of adjuvant treatment for early-stage breast cancer and Ductal Carcinoma in situ (DCIS), or from four weeks to 18 months post-surgery if no further adjuvant therapy was planned. Participants with DCIS were included as they are also likely to benefit from nurse-led shared care models. Additional eligibility criteria included the ability to speak and read English, being ambulatory at the time of recruitment, an Eastern Cooperative Oncology Group (ECOG) performance status of zero or one, the ability to nominate a GP or GP clinic for follow-up care, and access to a telephone. Potential participants were excluded if they had severe mental, cognitive, or physical conditions that limited their ability to provide informed consent. All participants provided written informed consent. Eligibility was determined by the research nurse and patients were invited to participate by their treating clinician before approached for consent by the research nurse.

### Randomisation and masking

Participants were allocated in a 1:1 ratio by a research assistant not involved in the trial, using a concealed computer-generated randomisation schedule, which had random permuted block sizes of eight and four, stratified by (1) surgery only, (2) surgery and radiation only, (3) surgery and chemotherapy ± radiation for Human Epidermal Growth Factor Receptor 2 (HER2)-negative breast cancer, and (4) surgery and chemotherapy ± radiation for HER2-positive breast cancer. Participants, and nurses involved in the intervention implementation, were aware of the allocated group as it was not possible to blind the participants or nurse administering the intervention due to the nature of the nurse-enabled consultation. However, researchers who assisted in data collection and conducted data analyses were blinded to group allocation.

### Procedures

Participants were recruited from outpatient oncology clinics at the PAH. Potentially eligible participants were identified by research nurses. All eligible participants provided written informed consent prior to being enrolled and randomised into the trial. Recruitment of 30 people per arm was ascertained *a priori* as sufficient to provide insights into the intervention feasibility and preliminary effectiveness.^17^ Treating medical specialists of participants were notified of their trial participation.

Participants randomised to the control arm received usual specialist-led follow-up care, which was non-standardised with activities and follow-up schedule dependent on the treating specialist (i.e., medical or radiation oncologist or surgeon). Additionally, participants in the control arm received a cancer survivorship information booklet “Living Well After Cancer” by the Cancer Council of Queensland.^18^ Australia's healthcare system combines public and private funding, with many services covered by the national health insurance (i.e., Medicare). GP consultations can be free if the GP bulk bills, though many practices charge a fee above the government rebate. Prescription medicines are co-paid by the patient, subsidized under a pharmaceutical benefits scheme. Specialist visits, allied health services, and community-based care often attract out-of-pocket costs, unless bulk-billed or subsidized through Medicare plans for chronic conditions. Public hospital care is free, while private hospital care involves significant costs, even with insurance.

Participants randomised to the intervention arm received the EMINENT intervention. EMINENT is a multi-faceted intervention that includes a pre-specified shared-care pathway for post-treatment follow-up. The key components of the intervention were 1) Specialist Breast Cancer Nurse consultation, 1a) Booster Nurse Consultation (offered to participants with Coronavirus Disease (COVID)-related delays in GP involvement greater than 3 months and up to 18 months), 2) Pharmacist Consultation, 3) Case Conference with GP, and 4) shared follow-up care arrangements. Details of these key components are presented in Supplementary Table S1. GPs were encouraged to use Medicare codes (directly from the government) to get reimbursed for their time for the case conference.

### Outcome measures—feasibility and process

Feasibility and process outcomes of implementing the EMINENT intervention were collected. For this study, feasibility refers to the practicality and viability of implementing it in a real-world healthcare setting. This was assessed by documenting the attendance rates of the specialist nurse-enabled consultation, GP case conference, pharmacist consultation, and the optional booster specialist nurse-enabled consultation, collectively by the specialist nurse and research staff. The completion rates of components within the specialist nurse-enabled consultation (including pre-clinic preparation required) and GP case conferences were also collected by the intervention nurse i.e., how long it took to run each session, the percentage of planned components within each that were implemented. For example, how long did each GP case conference took and whether rationale of treatment was explained or return to work booklet was provided during nurse-enabled survivorship consultation. Furthermore, open-ended responses from the patient satisfaction survey and contents of the survivorship care plan (SCP) were collected from the patients and specialist intervention nurse, respectively.

### Outcome measures—preliminary effectiveness

Preliminary effectiveness of the EMINENT intervention was assessed using patient-reported outcomes (i.e., health-related quality of life (HRQoL)) as part of paper or electronic surveys powered by were Research Electronic Data Capture (REDCap) and self-completed by the participants at: (t_1_) baseline, upon enrolment and prior to randomisation; (t_2_) 3-months, (t_3_) 6-months, and (t_4_) 12-months post-enrolment (Supplementary Table S2). The primary outcome, HR-QoL, was assessed using the Functional Assessment of Cancer Therapy-Breast Cancer (FACT-B), which assesses five domains of HRQoL as baseline, 3-, 6- and 12-months. Several secondary outcomes were also collected. Dietary behaviours (usual vegetable and fruit intake) were assessed with two short questions from the National Nutrition Survey. Physical activity and sedentary behaviour were assessed with the Active Australia Survey and a single question item from the International Physical Activity Questionnaire, respectively. HRQoL, diet, physical activity and sedentary behaviour were assessed at baseline, 3-, 6-, 12-months. Financial toxicity was assessed with the Comprehensive Score for Financial toxicity—Functional Assessment of Chronic Illness Therapy, at baseline, 6-, and 12-months. Patient experience of care was assessed with the Picker Patient Experience 15, at baseline, and that included seven dimensions of care including emotional support, coordination and physical comfort. Satisfaction of care was assessed with a 0–10 numerical analogue scale, with 0 being the least satisfied and 10 being the most satisfied at 12-months.

For both adverse events and health resource utilisation (unplanned hospital admission emergency department presentations and acute care service use, treating specialists, nurse clinician and allied health appointments from hospital records), the exploratory 24-months timepoint (t_5_) was selected as it was considered that such data would require a longer period to see potential differences, and that data could be collected through electronic medical record and would not increase patient burden. Data collection of health services utilisation outside the hospital was beyond the scope of the study due to the limited resources available to explore this context. Other clinical and demographic data that was collected at baseline were age, sex, education level, living arrangements, marital status, income, menopausal status, comorbidities, and breast cancer specific characteristics (diagnosis date, subtype, location, stage, and treatments completed).

### Statistics

Descriptive statistics for continuous variables were reported using means, standard deviations. R and Statistical Package for Social Sciences (SPSS) were used for data analyses. General estimating equations (GEE) were used to account for repeated measures to assess differences between control and intervention arms. Residuals of models were checked for assumptions and remediated appropriately. Data were log-transformed for linear models where residuals were not normally distributed, and back-transformed geometric means with 95% Confidence Intervals (CIs) were reported. The statistical model included time, arm, baseline scores, modality type (stratification variable), and the interaction between time and arm. Poisson regression was used to analyse fruit serves, and negative binomial regression was used to analyse Picker Patient Experience (PPE) due to over-dispersion. The number of specialty visits (medical oncologist, surgical oncologist, radiation oncologist, cancer nurse clinician, allied health, and other specialty) per patient over a 24-month period, and patient satisfaction scores were compared using Wilcoxon rank sum test between groups with medians and interquartile range reported.

The responses from the open-ended satisfaction surveys and the SCPs were content analysed. Two members of the research team (CYH, JL) reviewed the contents of the SCP independently, for key issues relevant to patients with cancer. Two members of the research team (CE, RJC), including one GP and one specialist cancer clinician assessed the goals and determined whether they fall within the role of a GP (or their referral to an allied health practitioner) to address in primary care. The contents were categorised within a set of common issues, such as exercise, diet, and mental well-being. Any discrepancies between the two researchers were resolved through a discussion and with a third member of the research team (CYH, JL, CE or RJC) when consensus was not reached.

### Role of funding source

The funding body had no role in the study design, its data collection, management, analysis, and interpretation; preparation, review, or approval of the manuscript; and decision to submit the manuscript for publication.

## Results

**Fig. 1:**
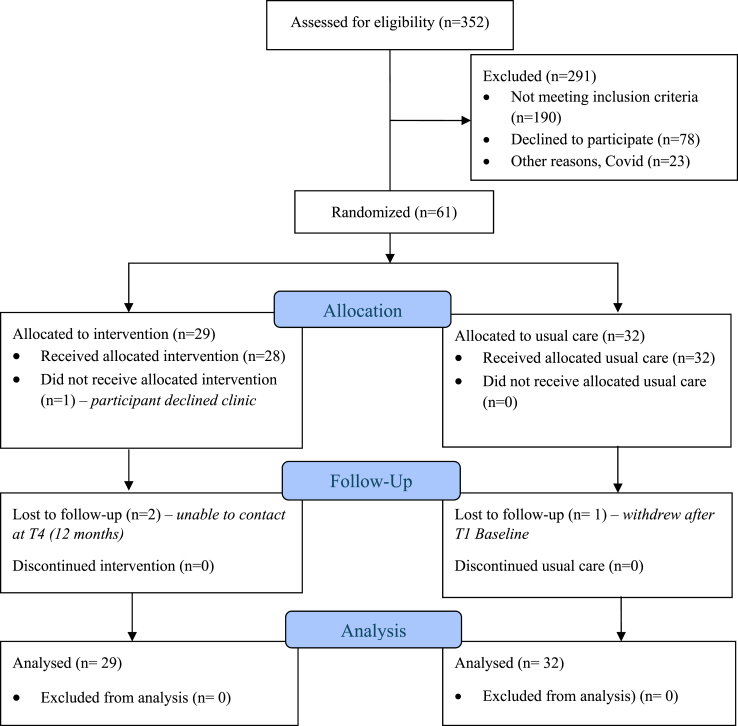
Overview of the EMINENT study with CONSORT Flow Diagram.

Of the 352 people assessed for eligibility, 190 were deemed ineligible. Out of the 162 that were eligible, 79 (49%) declined to participate, 23 (14%) were excluded for other reasons. Sixty-one (38%) people consented and were randomised to the intervention (n = 29) or control (n = 32) arms (Fig. 1) between 3rd December 2019 to 13th April 2021. There were no clinically relevant differences between groups, except body mass index (BMI) i.e., control group tended to have a higher BMI than intervention group and two lifestyle/sociodemographic factors i.e., three current smokers were present in the intervention group and more participants in control were living alone. Participants were all women with a mean age of 62.9 Standard Deviation (SD) = 10.9 years (see Table 1). Most participants were Caucasian (n = 51; 84%), had completed up to secondary level education (n = 41; 67%), and were married (n = 39; 64%). The most common cancer type was invasive ductal carcinoma (n = 38; 62%) followed by Ductal Carcinoma In Situ (DCIS) (n = 15; 25%), where most participants had Stage 1 disease at diagnosis (n = 28; 46%). All participants had breast surgery. Nine (control, n = 4; intervention, n = 5) had breast surgery only, without any adjuvant chemo and/or radiotherapy. Most participants had breast conserving surgery (n = 46; 75%) with adjuvant chemotherapy (n = 20; 33%) and radiation therapy (n = 53; 87%) and were treated with only one chemotherapy agent (n = 25; 41%). The two most common chemotherapy agents prescribed in this cohort were paclitaxel (n = 20; 33%) and cyclophosphamide (n = 17; 28%). A small proportion of participants (n = 6; 10%) received targeted therapy (i.e., monoclonal antibody) and about half of the participants (n = 32; 52%) were on hormonal therapies (i.e., selective estrogen receptor modulators, aromatase inhibitors or a luteinising hormone-releasing hormone agonist). Most participants had radiation therapy (n = 53; 87%) and were post-menopausal prior to treatment (n = 40; 66%). The most common comorbidities were chronic back pain (n = 21; 34%), hypertension (n = 14; 23%), osteoarthritis (n = 15; 25%) and depression (n = 11; 18%). Seven participants (11%) had prior diagnoses of cancer, with one (2%) participant with two previous cancers (gynaecological and bowel).

**Table 1:** Baseline characteristics of study participants (n = 61).

Characteristics^a^	Control (n = 32)	Intervention (n = 29)
Age, years, mean (SD)	61.5 (11.9)	64.4 (9.6)
Body Mass Index, kg/m^2^, mean (SD)	30.2 (6.1)	26.8 (4.3)
Waist circumference, cm, mean (SD)	93.3 (20.3)	86.9 (19.3)
Ethnicity		
Caucasian	28 (88)	4 (12)
Non-Caucasian	23 (79)	6 (21)
Education		
Up to primary	1 (3)	3 (10)
Up to secondary	22 (69)	19 (66)
Tertiary and above	9 (28)	7 (24)
Income (AUD)		
Up to 15,599	4 (12)	9 (31)
15,600–41, 599	10 (31)	6 (21)
41,600–77,999	7 (22)	6 (21)
78,000 and more	6 (19)	4 (14)
Prefer not to say	5 (16)	4 (14)
Living arrangement		
Living with partner	16 (50)	21 (72)
Living with other family member or friend	6 (19)	6 (21)
Alone	10 (31)	2 (7)
Marital status		
Married/De facto	17 (53)	22 (76)
Single/widowed/divorce	15 (47)	7 (24)
Current smokers	0 (0)	3 (10)
Cancer classifications		
Ductal carcinoma in situ	8 (25)	7 (24)
Lobular carcinoma in situ	0 (0)	1 (3)
Invasive ductal carcinoma	22 (69)	16 (55)
Invasive lobular carcinoma	2 (6)	4 (14)
Local advanced breast cancer	0 (0)	1 (3)
Stage of disease		
Stage I	12 (38)	16 (55)
Stage II	9 (28)	8 (28)
Stage III	4 (12)	0 (0)
Not available	7 (22)	5 (17)
Surgery type		
Breast conservative surgery	26 (81)	20 (69)
Unilateral mastectomy	4 (12)	7 (24)
Bilateral mastectomy	2 (6)	2 (7)
With adjuvant therapy	23 (72)	23 (79)
Chemotherapy	11 (34)	9 (31)
Targeted therapy	3 (10)	3 (10)
Hormonal therapy	15 (47)	17 (59)
With radiation therapy	29 (91)	24 (83)
Menopause prior to treatment^b^	19 (59)	21 (72)
Comorbidities		
Chronic back pain	9 (28)	12 (41)
Hypertension	9 (28)	5 (17)
Osteoarthritis	12 (38)	3 (10)
Depression	6 (19)	5 (17)
Type 2 Diabetes Mellitus	6 (19)	1 (3)
Previous diagnoses of cancer other than breast		
Skin	3 (9)	2 (7)
Gynaecological	1 (3)	1 (3)
Bowel	1 (3)	0 (0)

a Data expressed as mean and (standard deviation (SD)) for continuous variables; absolute numbers (percentage) for categorical variables. Groups were similar across most demographic and clinical variables; the control group tended to have higher Body Mass Index and waist circumference than the intervention group.

b Excludes perimenopause.

### Feasibility and process outcomes

Data on recruitment time from the last treatment/surgery are presented in Supplementary Table S3. Two participants were recruited outside of the permissible recruitment windows (63.0 days prior to last treatment was present in one case, and 22.0 days post-surgery in another) and were retained in the intention-to-treat (ITT) analyses. The intervention had 100% (28/28) attendance at both scheduled specialist nurse consultations and GP case conferences. For the specialist nurse survivorship consultation, the average time taken to complete was 82 (SD = 20.5) minutes with an average 35 (SD = 13.1) minutes for pre-consultation preparation and 47 (SD = 15.0) minutes in session with the patient. Individual elements of the nurse-enabled consultation preparation and delivery are available in Supplementary Figure S1. In preparing for the consultation session, medication information was recorded in the SCP for most participants with clarification of relevant information with the treatment team completed 82% (23/28) of the time. During the nurse-enabled survivorship consultation, explaining the rationale for treatment, goal setting, using motivational interviewing, exploring patient symptoms, and providing nurse contact details were completed 100% (28/28) of the time, while provision of a return-to-work booklet occurred in only 11% (3/28) of the sessions.

The median and interquartile range (IQR) time taken to complete the GP case conference was 15 (10, 20) minutes and ranged from 3 to 30 min. During the GP case-conference, the specialist cancer nurse checked the GP had received a copy of the SCP, discussed each team member's responsibilities, agreed for the GP to conduct physical exams, and discussed allied health referrals or resources in 100% (28/28) of consultations, with a discussion of the participants' symptom and self-management plan and involvement of the general practice nurse occurring in at least 95% (27/28) of consultations. Discussion around billing for patient appointments, GP involvement in an annual review of the SCP, and discussion of potential financial toxicity occurred in at least 80% (23/28) of consultations. Individual elements of the GP case conference are available in Supplementary Figure S2. All intervention participants received the pharmacist clinic component of the intervention. None of the participants attended the optional booster specialist nurse-enabled consultation.

At 6 months, complete follow-up data were available in 100% (29/29) and 97% (31/32) of the intervention and control arm respectively. At 12 months, complete follow-up data were available in 93% (27/29) and 97% (31/32) of the intervention and control arm respectively. Follow-up attrition rates at 6 months and 12 months were 2% (1/61) and 5% (3/61) respectively.

**Fig. 2:**
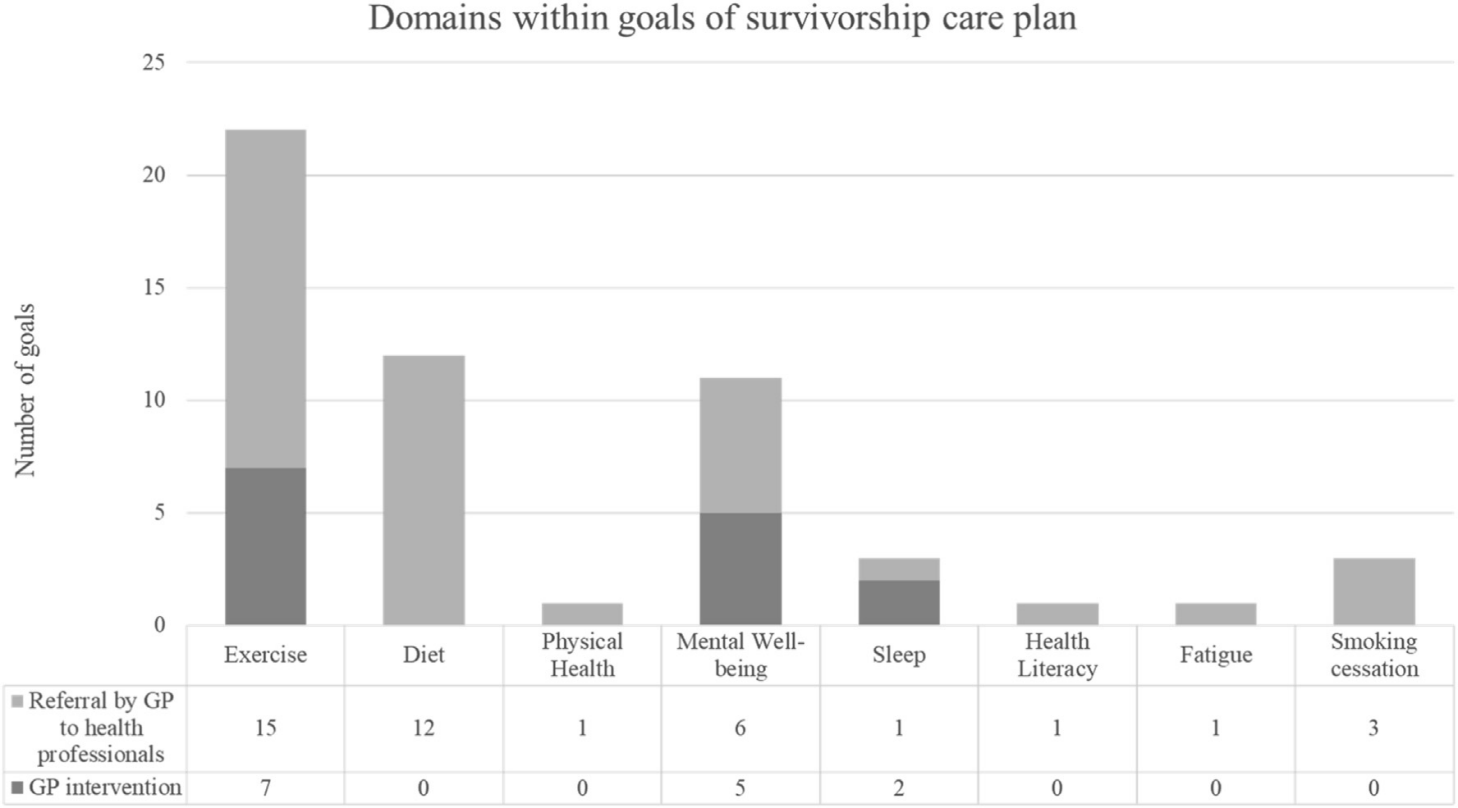
Domains within goals of survivorship care plan (n = 28).

Evaluation of the 29 SCPs developed for the intervention participants indicated the top three most common goals related to exercise (n = 23), diet (n = 12) and mental well-being (n = 11) (Fig. 2). All goals within the SCPs were determined to be goals that could be managed either solely by the GP or via GP referral to an allied health professional. Examples of illustrative quotes from the SCP goals that were common include: *“I would like to improve our diet as a family to help reduce health issues in the future”* (diet); “*I will focus on my mental health and access appropriate support*” (mental well-being); *“To engage in physical exercise 10–*15 mins *x 3 weekly and increase over time”* (exercise).

### Preliminary effectiveness

#### Patient-reported outcomes over 12 months

**Fig. 3:**
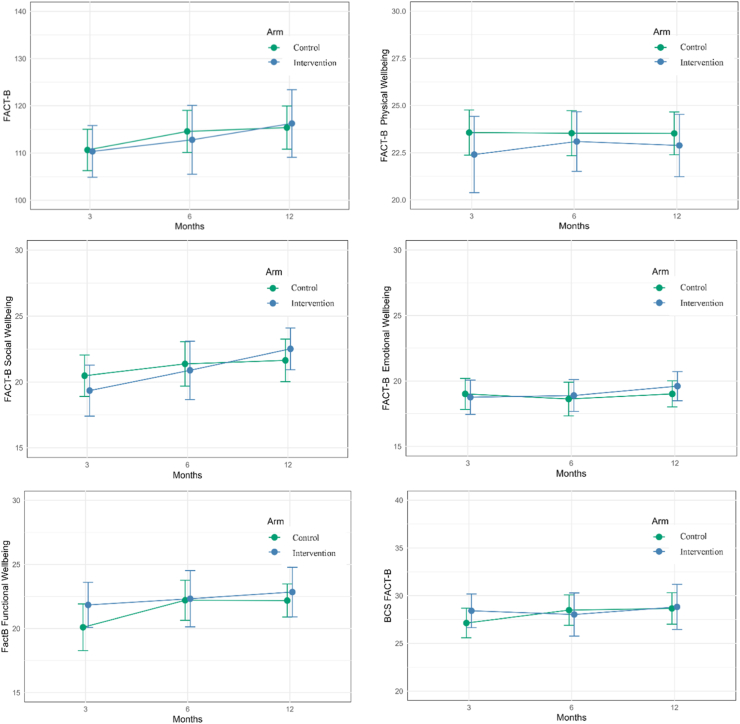
Adjusted mean FACT-B score (total and sub-scales) and 95% confidence intervals. Abbreviations: FACT-B, Functional Assessment of Cancer Therapy—Breast: consist of 37-items designed to measure five domains of Health-Related Quality of Life in breast cancer patients: Physical, social, emotional, functional well-being as well as a breast-cancer subscale. Score range from 0 to 123 points with lower scores indicating better health; BCS, Breast Cancer subscale.

There were no significant interactions between time and treatment arm for any patient-reported outcomes (P ≥ 0.05), nor were there significant group differences between treatment arms (P ≥ 0.05). Over time, patient-reported outcomes showed no significant changes, except for overall and Social FACT-B. Overall FACT-B scores tended to increase over time (P = 0.017), averaged over the group, 3-month FACT-B scores were 110.5 (CI: 106.6, 114.4) compared to 113.7 (CI: 109.1, 118.3, P = 0.208) at six months and 115.8 (CI: 111.4, 120.2, P = 0.013) at 12 months. This trend was also observed for Social FACT-B, with scores increasing over time (P = 0.009), at 3-months scores were 19.9 (18.6, 21.2) compared to 21.1 (CI: 19.7, 22.6, P = 0.161) at six months and 22.1 (CI: 20.9, 23.3, P = 0.007) at 12 months. Effects of intervention or control arm on outcome variables using ITT analyses are presented in Table 2, with the changes in FACT-B subscales between groups presented in Fig. 3. There were no differences in overall QoL or any QoL subscales between groups. There is no significance difference between groups for fruits and vegetables, and physical activity, though some trends favouring the intervention e.g., increasing fruits and vegetables intake and physical activity over time could be observed.

**Table 2:** Baseline and preliminary effects of intervention on outcome variables, intention-to-treat analyses.

Outcome variable	Control (n = 32)	Intervention (n = 29)	Control (n = 32)	Intervention (n = 29)	*Between-group difference or ratio*^a^
	Mean (SD)		Adjusted Mean (95% CI)		Mean differences (95% CI)
***FACT-B***^b^***—total***					
0 month, t_1_	107.8 (19.3)	111.1 (21.1)			
3 months, t_2_	108.0 (19.5)	109.0 (19.7)	110.6 (106.3, 115.0)	110.3 (106.3, 115.0)	−0.3 (−9.3, 8.7)
6 months, t_3_	112.0 (21.6)	111.4 (19.4)	114.6 (110.1, 119.0)	112.8 (105.5, 120.1)	−1.8 (−13.1, 9.6)
12 months, t_4_	112.8 (21.0)	115.8 (19.3)	115.4 (110.8, 119.9)	115.4 (110.8, 119.9)	0.9 (−11.0, 12.7)
***FACT-B***^b^***—physical wellbeing subscale***					
0 month, t_1_	22.8 (4.5)	22.8 (3.6)			
3 months, t_2_	24.0 (4.1)	22.5 (5.2)	23.6 (22.4, 24.8)	22.4 (20.4, 24.4)	−1.2 (−4.0, 1.7)
6 months, t_3_	23.9 (3.6)	23.2 (3.9)	23.5 (22.3, 24.7)	23.1 (21.4, 24.7)	−0.4 (−2.9, 2.0)
12 months, t_4_	23.9 (3.6)	23.0 (4.8)	23.5 (22.4, 24.7)	22.9 (21.2, 24.5)	−0.6 (−3.2, 2.0)
***FACT-B***^b^***—social wellbeing subscale***					
0 month, t_1_	20.5 (6.3)	22.2 (6.3)			
3 months, t_2_	19.2 (5.9)	19.1 (7.0)	20.5 (18.9, 22.0)	19.3 (17.4, 21.3)	−1.1 (−4.6, 2.3)
6 months, t_3_	20.1 (7.5)	20.6 (6.4)	21.4 (19.7, 23.1)	20.9 (18.7, 23.1)	−0.5 (−4.4, 3.4)
12 months, t_4_	20.4 (6.4)	23.1 (4.4)	21.6 (20.0, 23.3)	22.5 (20.9, 24.1)	0.9 (−2.2, 4.0)
***FACT-B***^b^***—emotional wellbeing subscale***					
0 month, t_1_	18.4 (4.1)	19.0 (4.2)			
3 months, t_2_	18.8 (3.8)	18.8 (4.4)	19.0 (17.8, 20.2)	18.8 (17.5, 20.1)	−0.3 (−2.6, 2.1)
6 months, t_3_	18.5 (4.1)	18.9 (3.8)	18.6 (17.3, 19.9)	18.9 (17.7, 20.1)	0.3 (−2.1, 2.7)
12 months, t_4_	18.8 (4.0)	19.7 (3.2)	19.0 (18.0, 20.0)	19.6 (18.5, 20.7)	0.6 (−1.4, 2.6)
***FACT-B***^b^***—functional wellbeing subscale***					
0 month, t_1_	18.6 (6.2)	21.0 (5.6)			
3 months, t_2_	18.8 (6.7)	21.1 (5.3)	20.1 (18.3, 21.9)	21.8 (20.1, 23.6)	1.7 (−2.0, 5.5)
6 months, t_3_	20.9 (6.2)	21.6 (5.4)	22.2 (20.6, 23.8)	22.3 (20.1, 24.5)	0.1 (−3.9, 4.1)
12 months, t_4_	20.8 (5.1)	22.2 (4.7)	22.2 (20.9, 23.5)	22.8 (20.9, 24.8)	0.7 (−2.8, 4.1)
***FACT-B***^b^***—breast cancer subscale***					
0 month, t_1_	27.6 (5.4)	26.0 (7.9)			
3 months, t_2_	27.3 (5.8)	27.4 (6.2)	27.1 (25.6, 28.7)	28.4 (26.7, 30.2)	1.3 (−1.9, 4.5)
6 months, t_3_	28.6 (5.7)	27.1 (7.2)	28.5 (26.9, 30.1)	28.0 (25.8, 30.3)	−0.5 (−4.2, 3.3)
12 months, t_4_	28.8 (5.8)	27.9 (7.0)	28.7 (27.0, 30.3)	28.8 (26.5, 31.2)	0.2 (−3.9, 4.2)
***COST-FACIT***^c^					
0 month, t_1_	31.0 (9.8)	30.9 (8.9)			
3 months, t_2_	31.0 (8.6)	31.2 (9.2)	31.2 (28.5, 34.0)	31.9 (1.7, 2.5)	0.6 (−4.2, 5.5)
12 months, t_4_	30.4 (9.1)	29.2 (9.8)	30.8 (28.3, 33.3)	30.3 (26.5, 33.3)	−0.6 (−5.9, 4.7)
	**Geometric mean (95% CI)**			**Ratio (95% CI)**	

***Walk time per week, minutes***					
0 month, t_1_	82.7 (43.5, 156.1)	74.1 (37.4, 146.1)			
3 months, t_2_	27.9 (12.1, 62.5)	82.2 (46.2, 145.8)	26.8 (11.8, 59.1)	84.1 (43.3, 162.3)	3.07 (0.83, 11.31)
6 months, t_3_	46.7 (20.8, 103.0)	52.6 (26.5, 103.6)	46.2 (22.8, 92.6)	53.8 (27.0, 106.3)	1.16 (0.32, 4.27)
12 months, t_4_	35.5 (15.4, 80.2)	41.7 (16.9, 100.6)	35.2 (16.5, 74.0)	39.9 (16.5, 94.7)	1.13 (0.23, 5.60)
***Total moderate to vigorous physical activity per week, minutes***^d^					
0 month, t_1_	151.3 (83.9, 272.7)	123.1 (68.9, 219.8)			
3 months, t_2_	101.5 (60.0, 171.7)	199.1 (124.2, 319.4)	51.2 (26.5, 98.8)	115.5 (61.6, 216.7)	2.26 (0.72, 7.10)
6 months, t_3_	259.5 (155.3, 433.6)	215.4 (127.5, 363.7)	101.5 (55.3, 186.3)	117.3 (62.6, 219.7)	1.16 (0.39, 3.42)
12 months, t_4_	183.7 (108.3, 311.5)	222.5 (140.1, 353.5)	87.3 (47.0, 162.0)	134.5 (72.5, 249.5)	1.54 (0.51, 4.65)
***Sitting time per week, minutes***					
0 month, t_1_	271.4 (215.3, 342.2)	301.3 (235, 386.3)			
3 months, t_2_	304.6 (226.4, 409.9)	221.5 (172.4, 284.5)	316.8 (242.8, 413.4)	254.8 (206.1, 314.9)	0.8 (0.49, 1.31)
6 months, t_3_	289.3 (223.3, 374.8)	309.6 (249.2, 384.7)	305.6 (250.0, 373.5)	349.0 (284.0, 428.7)	1.14 (0.76, 1.73)
12 months, t_4_	310.6 (262.4, 367.5)	291.1 (231, 367.0)	326.5 (286.5, 372.1)	309.7 (245.8, 390.3)	0.95 (0.65, 1.39)
***Total vegetables intake, servings per day***^e^					
0 month, t_1_	2.9 (2.4, 3.5)	2.4 (1.8, 3.2)			
3 months, t_2_	2.5 (1.8, 3.5)	2.8 (2.3, 3.5)	2.5 (1.8, 3.5)	3.0 (2.4, 3.7)	1.20 (0.72, 2.00)
6 months, t_3_	2.9 (2.2, 3.8)	3.2 (2.7, 3.8)	2.9 (2.3, 3.7)	3.4 (2.9, 4.1)	1.18 (0.80, 1.72)
12 months, t_4_	2.4 (1.7, 3.2)	2.6 (1.8, 3.8)	2.4 (1.7, 3.3)	2.8 (1.9, 4.0)	1.15 (0.61, 2.17)
***Total fruits intake, servings per day***^e^					
0 month, t_1_	1.9 (1.5, 2.4)	2.0 (1.6, 2.6)			
3 months, t_2_	1.9 (1.5, 2.5)	2.1 (1.6, 2.7)	2.0 (1.5, 2.5)	2.1 (1.7, 2.7)	1.09 (0.69, 1.73)
6 months, t_3_	1.7 (1.3, 2.3)	2.0 (1.5, 2.5)	1.8 (1.5, 2.2)	2.0 (1.7, 2.5)	1.13 (0.82, 1.56)
12 months, t_4_	2.0 (1.6, 2.6)	2.5 (2.0, 3.2)	2.1 (1.6, 2.6)	2.6 (1.9, 3.5)	1.24 (0.73, 2.10)

**Notes:** Data are presented as mean (Standard Deviation (SD)) or geometric mean and 95% confidence intervals. Outcomes were similar between all variables at baseline. The geometric mean and 95% CI were calculated using an intercept model with each group. The Tweedie model was used for Total moderate to vigorous physical activity and the Poisson model was used for Fruit. Otherwise, linear models were used.

**Abbreviations:** FACT-B, Functional Assessment of Cancer Therapy—Breast; COST-FACIT, COmprehensive Score for financial Toxicity-Functional Assessment of Chronic Illness Therapy.

a Adjusted estimates were derived from general estimating equation models adjusted by baseline and by stratification variable of treatment type.

b Functional Assessment of Cancer Therapy—Breast (FACT-B): consist of 37-items designed to measure five domains of Health-Related Quality of Life in breast cancer patients: Physical, social, emotional, functional well-being as well as a breast-cancer subscale. Score range from 0 to 123 points with lower scores indicating better health.

c Comprehensive Score for financial Toxicity-Functional Assessment of Chronic Illness Therapy (COST-FACIT): consist of 12 items: 1 financial item, 2 resource items and 8 affect items, and additional item on financial well-being. A lower score suggests worse financial toxicity.

d Total moderate to vigorous physical activity based on self-reported data from the International Physical Activity Questionnaire, a 27-item self-reported measure of physical activity.

e Total vegetables and fruits intake based on self-reported intake based on two short dietary questions from the National Nutrition Survey.

#### Satisfaction with care at 12 months

The median (IQR) scores for satisfaction with care at 12 months were 9.0 (6.0, 10.0) and 9.5 (8.0, 10.0) for control and intervention groups respectively and results from Wilcoxon rank sum tests showed no statistical difference (P = 0.37) between groups.

#### Healthcare utilisation in the hospital at 24 months (exploratory)

**Fig. 4:**
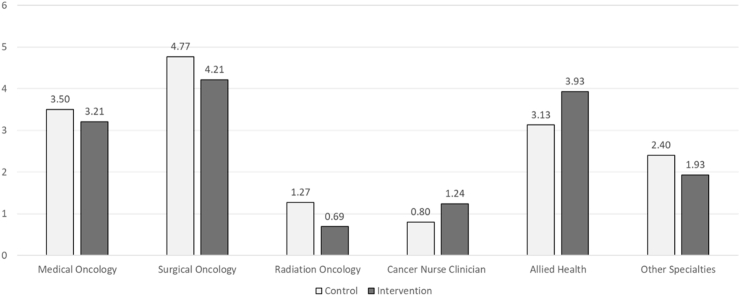
Number of average appointments attended per patient from baseline to 24 months (based on available data of control, n = 30; intervention, n = 29).

Healthcare utilisation data were available for 59 participants (control, n = 30; intervention, n = 29) for 24 months upon enrolment to study. There was a total of 476 and 441 appointments (medical oncology, surgical oncology, radiation oncology, cancer nurse clinician (from either medical, surgical or radiation oncology), allied health, and other specialist) for the control and intervention group, respectively. Fig. 4 shows the average appointments attended per patient from baseline to 24-months. There were fewer total average number of radiation oncology appointments in the intervention as compared to the control group (intervention = 0.69 versus control = 1.27, P = 0.013). There were also more total average number of cancer nurse clinician (intervention = 1.24 versus control = 0.80, P = 0.026) and allied health (intervention = 3.93 versus control = 3.13, P = 0.029) appointments in intervention as compared to the control group. The number of unplanned hospital presentations at 24-months were low across both the intervention group (n = 7) and the control group (n = 4).

#### Adverse events at 24 months (exploratory)

There were no adverse events or deaths due to the intervention (i.e., injuries or medical events due to the trial that result in medical attention or restriction of daily living activities for more than two days) as documented or reported to ethics. Cancer recurrence data at 24-months (t_5_) was available for 58 participants. There was a report of a cancer (recurrent metastatic breast cancer) in the control group within 24-months (t_5_).

## Discussion

This study demonstrated that both the specialist nurse-enabled shared-care model and the trial are highly feasible in DCIS and stage I-III breast cancer survivors. A key contributor to this positive outcome lies within the role of the specialist nurse in bridging the gap in communication between health care providers and their patients through a combination of a structured SCP, specialist nurse-enabled consultation and GP case conference. To our knowledge, this study is the first to provide details of implementation within such a model of care in this population e.g., time taken for each component of the intervention, components within SCP, patient goals. The study also highlighted high level of congruence between goals set by cancer survivors in their SCP and the role of GPs, specialist nurses and allied health professionals (particularly in areas of exercise, diet, and mental well-being) in this population.

Poor communication between health care providers and between health care providers with their patients, as well as a lack of time and standardized protocol to complete the SCP were common barriers to implementation of a shared-care model in cancer survivorship as highlighted in a recent overview of systematic reviews regarding implementation of different models of post-treatment cancer survivorship care compared to specialist-led models.^10^ The present study demonstrated that the EMINENT intervention, consisting of a dedicated session led by a specialist nurse to gather the required information using a structured SCP, effectively enabled the nurse to act as a “conduit” to facilitate survivorship care across settings.^19^ During the specialist nurse-enabled consultation, initial drafting of the SCP prepared by the specialist nurse and their patients laid the ground work in mapping of care required post-treatment. Having a draft plan prior to GP case conference allows a more efficient use of GP time while still incorporating their input. More importantly, this strategy allowed GPs’ access to a succinct, easy-to-read summary of the SCPs for the continuity of shared care in the community setting (i.e., suggested referral to relevant allied health professionals, cancer recurrence surveillance, and more). Access to shared-care records in the form of a SCP populated by both patient and provider ensured availability of current and consistent information to all.

Having a dedicated and trained specialist nurse to facilitate the initial development of the SCP may explain the difference between the 100% completion rate of SCPs in this study compared to lower rates (5–54%) reported in other studies.^20^ The synergy of components within the EMINENT intervention (i.e., nurse-enabled consultation and GP case conference) might have improved clarity of GP roles and responsibilities, which is one of the facilitators identified towards implementation of shared-care models for cancer survivorship.^21^ Another reason for such high completion rates for the SCPs could also be the use of a structured survivorship care plan and protocol during the nurse-enabled consultation. Following a structured protocol embedded within the intervention enables continuity and consistency of care between a team of specialist nurses. It also reduces the threat of unsustainability of such a shared-care model during staff turnover.^15^

While the shared-care model may not be suitable for all, it is an appropriate alternative for many cancer survivors of various cancer types.^22^ In spite of the variations in shared-care models between health systems,^23^ the role of the GP and specialist nurse are undeniably essential to the successful implementation of shared care in cancer survivorship.^24^^,^^25^ The involvement of GPs and specialist nurses in the community allows the treating medical specialist more time for patients that are newly diagnosed, on active treatments, or have more complex cancers.^26^ Therefore, transitioning some aspects of cancer survivorship care from acute to community care (i.e., GPs and specialist nurses) can help alleviate the increasing burden of survivorship care to any acute care systems, without delivering care with comparative effectiveness on health outcomes (e.g., health-related quality of life) in this population.

The specialist nurse-enabled consultations and GP case conferences were important components of the EMINENT intervention as communication of patients’ concerns and goals were identified and recorded in the SCPs, which was then directly communicated to their respective GP for follow-up recommendations. Once patient-centred goals were identified and communicated, supportive care interventions addressing those concerns were able to be facilitated by their GPs. The GP case conference might also have improved clarity to responsibilities and roles of the GP and specialist nurse in supportive care, disease recurrence surveillance and prevention. Particularly, the use of allied health in the hospital by the intervention group was lesser. This may be because the GP has referred them back to the community for community allied health—which is a good direction towards sustainability for the shared-care model.

In this study, the top three most common goals of breast cancer survivors pertained to exercise, diet, and mental well-being. This may explain the higher cancer nurse clinician and allied health visits in the intervention group, as reflected in the areas of care needs within the survivorship care plan i.e., exercise, diet and mental well-being. These goals could be facilitated and managed by the GPs themselves (rather than an oncologist) or by GP referral and co-management with an allied health professional (e.g., exercise physiologist, dietitian, psychologist, or mental health worker). A recent review highlighted that medical and nursing professionals understand that referral to dietary and exercise professionals form part of their role in cancer care.^27^ Further, numerous tasks highlighted within survivorship care in this study fall under the scope of practice of GPs and specialist nurses. This meant that these tasks are not necessarily required to be completed by treating medical specialists, who should be better positioned to look after new patients. Further information on GPs’ level of comfort in managing common side effects from hormone therapy could also be explored.

One of the key resources to successful implementation of the EMINENT intervention was nursing time. Approximately 98 min (∼82 with specialist nurse and patient; ∼16 with specialist nurse and GP) were needed to complete the survivorship care planning process (i.e., identify care needs with patient, recording and communicating across clinical settings consisting of treating medical specialists and GPs). Planning is fundamental to the clinical care for any long-term conditions and multidisciplinary treatment planning is not new to cancer care.^28^^,^^29^ In this aspect, specialist nurses play a critical role in bridging silos between health systems and preventing fragmentation of acute to long-term cancer care.^30^ Within a nurse-enabled, share care cancer survivorship model, the value brought by specialist nurse consultation with patients and the wider multidisciplinary team and GPs extends beyond the time invested.^31^ Patients further from treatment may require follow-up addressing late effects, while those that recently completed treatment might prioritize immediate physical recovery and psychosocial support. The nurse-enabled consultation considers the patients’ time since treatment completion and addressed individual care needs using the survivorship care plan during the 1-h nurse consultation as part of the intervention.

The exploratory analysis of clinic appointments at 24 months revealed a significantly lower radiation oncology attendance in the shared-care arm. In the post-treatment setting, these appointments focused on surveillance and provision of general support. We postulate that this may be due to the increased confidence of the radiation oncologist in the shared care received by the patients, and reduced the requirement of follow up. An economic evaluation from a health service perspective may confirm that all relevant resourcing between specialist-led and shared-care, bearing in mind that previous shared-care models in prostate and colorectal cancers have showed equivalent QoL with lower costs.^13^^,^^14^

The above proposed resources discussed for the successful implementation of a specialist nurse-enabled shared-care model in DCIS and stage I-III breast cancer survivors will vary between different health care systems. For such model to be used on a large scale, a systems-thinking approach using a systems-level framework such as the World Health Organisation system building blocks may be useful to identify and understand relevant system-level factors related to shared-care practices, and come up with strategies that leverage existing synergies and create or promote new relationships between various system-level factors within that particular health system.^27^

A limitation of this study was that it was conducted during the height of the COVID-19 pandemic. While potential participants could not participate as illustrated earlier in Fig. 1, delivery of the intervention was impacted minimally as patient could still be contacted via telephone and an additional Booster Nurse Consultation was also offered to participants with COVID-related delays in GP involvement. Although the shortest GP consultation meeting was 3 min, the GP was provided a comprehensive care plan, and the meeting allowed for an opportunity for GPs to clarify any questions. Some GPs may feel more confident in managing patients with breast cancer than others, and the SCPs might have also been sufficiently detailed so no questions were raised. Future studies are needed to explore these factors as well as GPs acceptability of preferred engagement methods. Also, this was a pilot study and was not powered sufficiently to measure its impact on specific health outcomes. The results may only represent patients with early-stage breast cancer and cannot be extrapolated to assume an improvement on hospital and economical outcomes on metastatic breast cancer. Though time taken for specialist nurse-enabled consultation and GP conferences were captured, data on GP consultation time following those interventions and health services utilization in the primary care setting were not collected. The large recruitment window of four to 18 months post-surgery or adjuvant treatment, while reflects the pragmatic nature of women requiring the necessary, coordinated survivorship support, it presents a challenge as it adds to the heterogeneity of the trial participants. Future definitive, statistically powered, large implementation effectiveness trials should include this wide window with practical stratification of timeframe post treatment (e.g., <3 months; 3–12 months; beyond 12 months). Defining the end of treatment time point needs to be pragmatically and functionally determined given the ever-evolving treatment landscape. The authors also acknowledged the debate around different treatment approaches and expect that future research with larger sample sizes may examine the effectiveness of this intervention on different cancer stages and treatments. A larger, statistically powered, hybrid effectiveness-implementation trial would be needed to confirm the comparative effectiveness presented here, and for further larger scale process evaluation (e.g., time taken for SCPs). The inclusion of a cost-effectiveness analysis could further support its implementation and uptake by existing cancer survivorship health services.

A larger Phase III-IV hybrid effectiveness-implementation study addressing these limitations is currently underway—Implementation of a nurse-enabled, shared-care follow-up model for early breast cancer survivors (The IBIS-Survivorship Study, ACTRN12621000188831).

Nurse-enabled shared care using the EMINENT intervention is feasible for DCIS and stage I-III breast cancer survivors in Australia. The EMINENT intervention provides comprehensive, person-centred survivorship care for this population by leveraging the skills of specialist nurses and GPs in the community and may reduce the burden on treating medical specialist and the acute care system.

## Contributors

RC and JE conceptualised the study. RC, SM, KC, DM, EM, JE, MR, CC, RP, MC and LT developed the methodology, sought and obtained funding and provided resources for the study. FCW, BC, MG, JR, RJ and LT administered and supervised the project. FCW, LT and CYH curated the data. LJ, CYH and JL performed formal analysis. RJC, FCW, CYH, JL, LJ, CE have verified the underlying data. RC, CYH, JL, CE, NH and AC interpreted the data. RC, FCW and CYH wrote the first draft of the paper. All authors have reviewed and agreed to the published version of the manuscript.

## Data sharing statement

De-identified data, participant information and consent forms is available from the corresponding author upon reasonable request.

## Declaration of interests

The authors declare that they have no competing interests.

## References

[1] Sung H., Ferlay J., Siegel R.L. (2021). Global cancer statistics 2020: globocan estimates of incidence and mortality worldwide for 36 cancers in 185 countries. CA A Cancer J Clin.

[2] AIHW A.G., Australian Institute of Health and Welfare (2022). Cancer Data in Australia.

[3] Moore H.C. (2020). Breast Cancer Survivorship. Semin Oncol.

[4] Schmidt M.E., Wiskemann J., Steindorf K. (2018). Quality of life, problems, and needs of disease-free breast cancer survivors 5 years after diagnosis. Qual Life Res.

[5] Fortin J., Leblanc M., Elgbeili G., Cordova M.J., Marin M.-F., Brunet A. (2021). The mental health impacts of receiving a breast cancer diagnosis: a meta-analysis. Br J Cancer.

[6] Lovelace D.L., McDaniel L.R., Golden D. (2019). Long-term effects of breast cancer surgery, treatment, and survivor care. J Midwifery Wom Health.

[7] Runowicz C.D., Leach C.R., Henry N.L. (2016). American cancer society/american society of clinical oncology breast cancer survivorship care guideline. CA Cancer J Clin.

[8] Cancer Council Victoria and Department of Health Victoria (2021).

[9] Cancer Australia (2016).

[10] Chan R.J., Crawford-Williams F., Crichton M. (2021). Effectiveness and implementation of models of cancer survivorship care: an overview of systematic reviews. J Cancer Surviv.

[11] Grunfeld E., Levine M.N., Julian J.A. (2006). Randomized trial of long-term follow-up for early-stage breast cancer: a comparison of family physician versus specialist care. J Clin Oncol.

[12] Giles C., Nehill C., Milch V., Zorbas H. (2014). Shared follow-up care for early breast cancer-results from an Australian national demonstration project. BMC Health Serv Res.

[13] Emery J.D., Jefford M., King M. (2017). Procare trial: a phase ii randomized controlled trial of shared care for follow-up of men with prostate cancer. BJU Int.

[14] Jefford M., Emery J.D., Martin A.J. (2023). Score: a randomised controlled trial evaluating shared care (general practitioner and oncologist) follow-up compared to usual oncologist follow-up for survivors of colorectal cancer. eClinicalMedicine.

[15] Lisy K., Kent J., Piper A., Jefford M. (2021). Facilitators and barriers to shared primary and specialist cancer care: a systematic review. Support Care Cancer.

[16] Chan R.J., Downer T.-R. (2018).

[17] Chan R.J., Emery J., Cuff K. (2020). Implementing a nurse-enabled, integrated, shared-care model involving specialists and general practitioners in breast cancer post-treatment follow-up: a study protocol for a phase ii randomised controlled trial (the eminent trial). Trials.

[18] Cancer Council Australia (2018).

[19] Lisy K., Kent J., Dumbrell J., Kelly H., Piper A., Jefford M. (2020). Sharing cancer survivorship care between oncology and primary care providers: a qualitative study of health care professionals' experiences. J Clin Med.

[20] Hill R.E., Wakefield C.E., Cohn R.J. (2020). Survivorship care plans in cancer: a meta-analysis and systematic review of care plan outcomes. Oncol.

[21] Fox J., Thamm C., Mitchell G. (2022). Cancer survivorship care and general practice: a qualitative study of roles of general practice team members in Australia. Health Soc Care Community.

[22] Jefford M., Chan R.J., Emery J.D. (2024). Shared care is an appropriate model for many cancer survivors. J Clin Oncol.

[23] Halpern M.T., Viswanathan M., Evans T.S., Birken S.A., Basch E., Mayer D.K. (2015). Models of cancer survivorship care: overview and summary of current evidence. J Oncol Pract.

[24] Elizondo Rodriguez N., Ambrosio L., La Rosa-Salas V., Domingo-Osle M., Garcia-Vivar C. (2022). Role of the nurse in the design, delivery, monitoring and coordination of cancer survivorship care plans: an integrative review. J Adv Nurs.

[25] Crabtree B.F., Miller W.L., Howard J. (2020). Cancer survivorship care roles for primary care physicians. Ann Fam Med.

[26] Sandell T., Schütze H., Miller A., Ivers R. (2023). Patients' acceptance of a shared cancer follow-up model of care between general practitioners and radiation oncologists: a population-based survey using the theoretical framework of acceptability. BMC Prim Care.

[27] Joseph R., Hart N.H., Bradford N. (2024). Adopting a systems-thinking approach to optimise dietary and exercise referral practices for cancer survivors. Support Care Cancer.

[28] Lhussier M., Eaton S., Forster N., Thomas M., Roberts S., Carr S.M. (2015). Care planning for long-term conditions–a concept mapping. Health Expect.

[29] Prabhu Das I., Baker M., Altice C., Castro K.M., Brandys B., Mitchell S.A. (2018). Outcomes of multidisciplinary treatment planning in us cancer care settings. Cancer.

[30] Ullgren H., Kirkpatrick L., Kilpeläinen S., Sharp L. (2017). Working in silos?–head & neck cancer patients during and after treatment with or without early palliative care referral. Eur J Oncol Nurs.

[31] Torreggiani M., Maselli D., Costi S., Guberti M. (2024). Models of care in providing comprehensive healthcare on cancer survivors: a scoping review with a tidier checklist analysis. Int J Environ Res Public Health.

