# Supplementary material for: Implementing a nurse-enabled, integrated, shared-care model involving specialists and general practitioners in early breast cancer post-treatment follow-up (EMINENT): a single-centre, open-label, phase 2, parallel-group, pilot, randomised, controlled trial
Source: eClinicalMedicine. 2025 Feb 5;81:103090. doi: 10.1016/j.eclinm.2025.103090 (PMC11848094; doi:10.1016/j.eclinm.2025.103090)
Supplement: Supplementary Figures and Tables [file mmc1.pdf]

Table S1 Five key components of the EMINENT model of care intervention

Key components	Personnel involved	Specific activities
**1. Nurse clinic** (30-60min) *Mode: face-to-face or telehealth*	Specialist Cancer Nurse[a] and patient	• Treatment summary<br>• Survivorship care planning<br>• Collaborative planning for health goals<br>• Post-treatment education
**1a. Booster Nurse Clinic[b]** (10–30 min) *Mode: face to face or telehealth*	Specialist Cancer Nurse[a] and patient	• Survivorship care planning<br>• Collaborative planning for health goals
**2. Pharmacist Consultation[c]** (20 min) *Mode: face to face or telehealth*	Cancer Pharmacist and patient	• Medication reconciliation<br>• Medication education
**3. Case conference with General Practitioner (GP)** (5–30 min) *Mode: teleconference*	Specialist Cancer Nurse[a] and GP (± one more healthcare team member)	• Nurse presents treatment summary and survivorship care plan<br>• Follow-up responsibilities of the GP negotiated<br>• GP questions answered<br>• Additional education and support provided to other healthcare team members (e.g., Practice Nurse)
**4. Shared follow-up care** *Mode: face to face or telehealth*	Cancer specialist, GP, and Specialist Cancer Nurse[a] and patient	• Cancer specialist reviews patient, orders mammogram, and completes full physical examination every 6 months for 2 years post-diagnosis, then every 12 months up to 5 years post-diagnosis<br>• GP reviews patient as per survivorship care plan at least every 12 months (e.g., for general health and comorbidity management, chronic disease management planning, psychosocial screening, management of cancer treatment toxicities and cancer-related symptoms, allied health referrals)<br>• GP contacts nurse interventionist with any patient concerns

[a]Medical Oncology Clinical Nurse Consultant, Breast Care Nurse, or McGrath Breast Care Nurse
[b]Offered to patients who have delays in GP involvement greater than 3 months (up to 18 months)
[c]Offered to patients who have completed chemotherapy and/or are scheduled to receive aromatase inhibitor, or have completed surgery and radiotherapy
Abbreviations: EMINENT, nurse-enabled, integrated, shared-care model involving specialists and general practitioners in early breast cancer post-treatment; GP, General Practitioner

Table S2: Outcome variables measured for all study participants in the EMINENT trial to measure patient level outcomes

Outcome	Scale/collection method	Description	Time point
Health-Related Quality of Life (HRQoL)	Functional Assessment of Cancer Therapy – Breast Cancer[39]	A 37-item instrument that measures five domains of HRQoL in breast cancer patients – physical, social, emotional, functional well-being and a breast-cancer subscale.	t_1, t_2, t_3, t_4
Dietary behaviors (usual vegetable and fruit intake)	National Nutrition Survey[41] (2 short dietary questions)	Both questions discriminate between groups with significantly different fruit and vegetable intakes. Information about which foods are included as vegetables and fruits is provided and serve sizes were described during administration	t_1, t_2, t_3, t_4
Physical activity	Active Australia Survey[42]	Designed to measure participation in leisure-time physical activity e.g., walking, moderate to intense exercise, gardening time	t_1, t_2, t_3, t_4
Sedentary behaviour	International Physical Activity Questionnaire[43]	A single question item to measure self-reported sedentary time (i.e., sitting time).	t_1, t_2, t_3, t_4
Financial Toxicity	Comprehensive Score for financial Toxicity-Functional Assessment of Chronic Illness Therapy (COST-FACIT)[44]	A set of 12 items: 1 financial item, 2 resource items and 8 affect items, and additional item on financial well-being. A lower score suggests worse financial toxicity	t_1, t_3, t_4
Patient experience of care	Picker Patient Experience 15[40]	A set of 15 questions considering 7 dimensions of care – respect, coordination, information/ communication/education, physical comfort, emotional support, involvement of relatives, and transitions to community	t_1
Satisfaction of care	Numerical analogue scale	0–10 numerical analogue scale with 0 being the least satisfied and 10 being the most satisfied.	t_4
Health service utilisation	Electronic medical records	Number of appointments attended – medical oncologist, radiation oncologist, radiation oncologist, other treating medical specialists, nurse clinician, allied health; Number of unplanned hospital presentations	t_5
Safety/Adverse events	Electronic medical records	Incidence of death, number of cancer recurrence	t_5

t_1, baseline; t_2, 3-month; t_3, 6-month; t_4, 12-month; t_5, 24-month

Abbreviations: EMINENT, nurse-enabled, integrated, shared-care model involving specialists and general practitioners in early breast cancer post-treatment; HR-QoL, Health-Related Quality of Life; COST-FACIT, Comprehensive Score for financial Toxicity-Functional Assessment of Chronic Illness

Table S3 Number of days from last treatment/surgery to recruitment

**Day since last treatment (surgery, chemo- and/or radiation therapy), median (Interquartile Range (IQR)); range of time intervals**			
	**Control (n=32)**	**Intervention (n=29)**	**Total (n=61)**
Total (Surgery with adjuvant chemo- and/or radiation therapy group; Surgery only group)	0.0 (-6.8 to 28.8); -63.0[a] to 164.0	1.0 (-5.0 to 48.0); -11.0 to 207.0	1.0 (-6.0 to 39.0); -63.0[a] to 207.0
	**Control (n=28)**	**Intervention (n=24)**	**Total (n=52)**
Surgery with adjuvant chemo- and/or radiation therapy group	-0.5 (-7.8 to 13.8); -63.0[a] to 146.0	-1.0 (-5.8 to 28.8); -11.0 to 98.0	-1.0 (-6.8 to 13.8); -63.0[a] to 146.0
**Days since surgery, median (IQR); range of time intervals**			
	**Control (n=32)**	**Intervention (n=29)**	**Total (n=61)**
Total (Surgery with adjuvant chemo- and/or radiation therapy group + surgery only group)	100.0 (73.3 to 209.5); 32.0 to 309.0	98.0 (70.5 to 191.5); 22.0[b] to 328.0	98.0 (71.5 to 195.0); 22.0[b] to 328.0
	**Control (n=28)**	**Intervention (n=24)**	**Total (n=52)**
Surgery with adjuvant chemo- and/or radiation therapy group	104.0 (78.8 to 226); 52.0 to 309.0	101.0 (73.0 to 177.0); 53.0 to 328.0	103.0 (76.3 to 205.0); 52.0 to 328.0
	**Control (n=4)**	**Intervention (n=5)**	**Total (n=9)**
Surgery only group	66.5 (34.8 to 145.5); 32.0 to 164.0	61.0 (32.5 to 205.0); 22.0[b] to 207.0	61.0 (37.5 to 183.5); 22.0[b] to 207.0

[a]63.0 days prior to last treatment was present in one case, with the next closest time interval being 26 days (within 4 weeks) prior to last chemo- or radiation therapy treatment. [b]22.0 days post-surgery was present in one case, with the next closest time interval being 32 days (after 4 weeks) after surgery. Overall, control group tended to have a lower range of recruitment since last surgery, chemo- or radiation therapy as compared to intervention group. Groups were similar across median time for days since surgery from recruitment.
Abbreviations: IQR, Interquartile Range

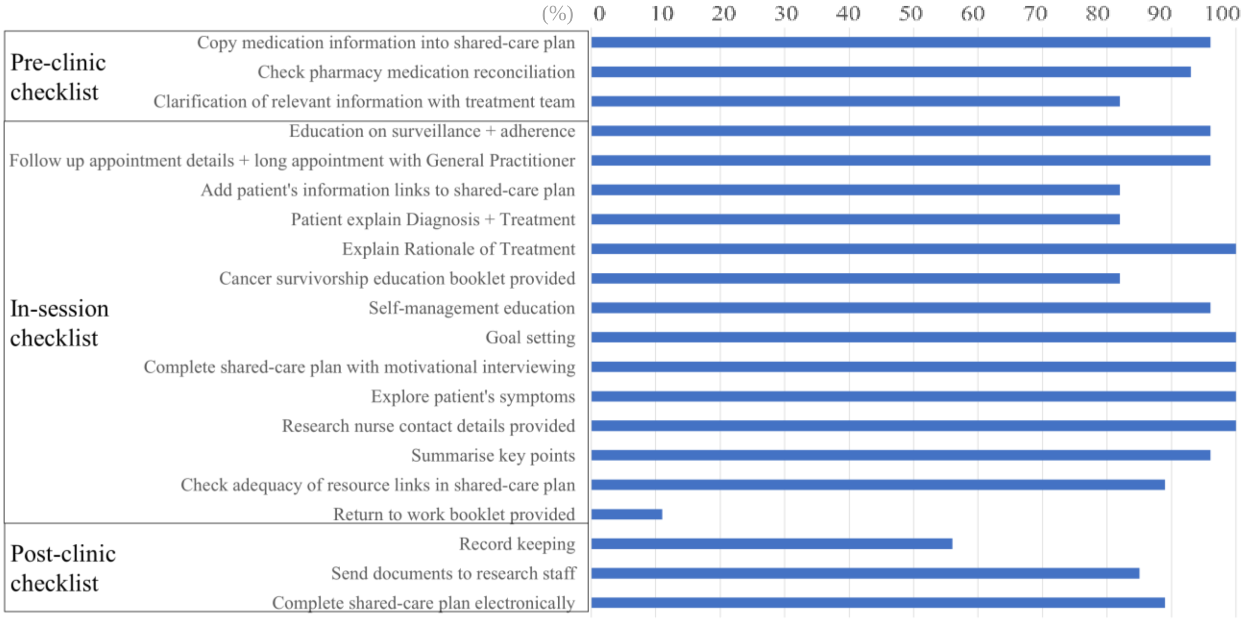

Figure S1. Completion rate of nurse-enabled survivorship consultation checklist (n=28)

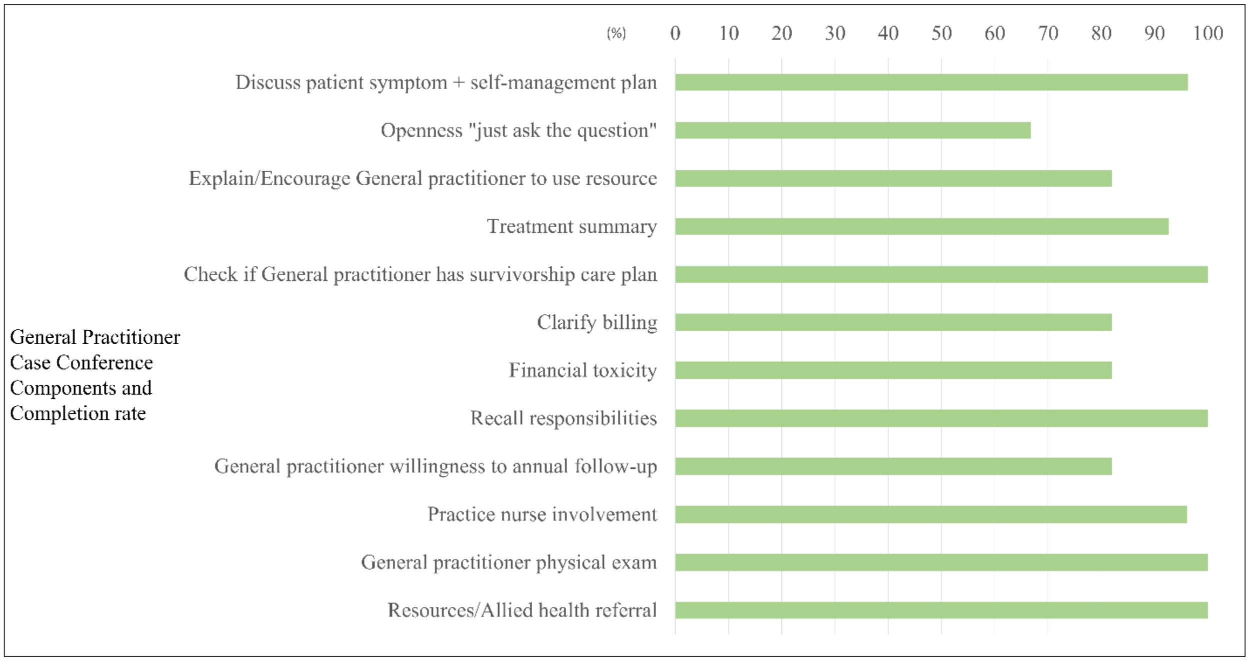

Figure S2. Completion rate of General Practitioner Case Conference checklist (n=28)
